# Immune-Mediated Demyelinating Polyradiculoneuropathies Linked to Systemic Lupus Erythematosus (SLE): A Systematic Review of Clinical Features, Diagnostic and Treatment Modalities

**DOI:** 10.7759/cureus.106675

**Published:** 2026-04-08

**Authors:** Abhishek Vadher, Swati Baraiya, Utsav Thakkar, Apoorv Tiwari, Kesava Manikanta Achuta, Sujata Kambhatla

**Affiliations:** 1 Internal Medicine, Garden City Hospital, Michigan State University, Garden City, USA; 2 Family Medicine, Bombay Hospital and Medical Research Center, Mumbai, IND; 3 General Medicine, Vedant Multispeciality Hospital, Ahmedabad, IND; 4 Internal Medicine, Garden City Hospital, Garden City, USA

**Keywords:** aidp, cidp, demyelinating polyneuropathies, gbs, sle

## Abstract

Immune-mediated demyelinating polyradiculoneuropathies-acute inflammatory demyelinating polyneuropathy (AIDP)/Guillain-Barré syndrome (GBS) and chronic inflammatory demyelinating polyneuropathy (CIDP)-are rare but potentially life-threatening manifestations reported in association with systemic lupus erythematosus (SLE). Evidence is scattered, and optimal diagnostic and therapeutic strategies remain uncertain. We performed a Preferred Reporting Items for Systematic Reviews and Meta-Analyses (PRISMA)-guided systematic review of PubMed, Embase, Cochrane Library, Scopus, and Google Scholar to identify English-language studies describing AIDP/GBS or CIDP occurring in patients with SLE and reporting clinical features, diagnostic findings, treatments, and outcomes. A total of 2,121 articles were yielded. After removal of duplicates, titles/abstracts were screened, full texts were assessed using predefined eligibility criteria, and data were extracted using a standardized form. Four studies met the inclusion criteria for qualitative synthesis. Across AIDP/GBS cohorts, there was a marked female predominance and a frequent “neuropathy-first” pattern, with AIDP/GBS preceding formal SLE diagnosis in more than half of reported cases. Clinically, acute limb weakness was the most common presentation, often accompanied by sensory symptoms. Cranial nerve involvement and respiratory involvement were also seen in many patients. Similarly, CIDP demonstrated strong female predominance and had variable temporal association with SLE, presenting as an initial, concomitant, or delayed manifestation during the course of SLE. Diagnostic evaluations consisted of cerebrospinal fluid studies and nerve conduction study; albuminocytologic dissociation and demyelinating features were supportive but not uniformly present across CIDP reports. Antiganglioside antibodies were usually negative in the tested patients. Treatments consisted of combined standard immunomodulatory approaches such as intravenous immunoglobulin (IVIg) and/or plasma exchange (PLEX) with lupus-directed immunosuppression (high-dose corticosteroids and cyclophosphamide). Outcomes were generally favorable in AIDP/GBS but had heterogeneous functional recovery in CIDP, particularly when therapy was delayed.

## Introduction and background

Systemic lupus erythematosus (SLE) is a complex autoimmune disease capable of affecting nearly any organ system, with clinical heterogeneity that necessitates standardized frameworks for consistent case identification in research and comparative studies. The 2019 European League Against Rheumatism/American College of Rheumatology (EULAR/ACR) classification criteria reflect modern thinking about SLE as an autoantibody-driven disease and provide a validated approach for classifying patients for research purposes [1].

Neurological involvement is a major contributor to morbidity in SLE and includes both central and peripheral nervous system syndromes. To support uniform reporting and improve diagnostic agreement in research, the American College of Rheumatology developed standardized nomenclature and case definitions for 19 neuropsychiatric syndromes in SLE, along with recommendations for minimum laboratory and imaging evaluation [2]. Despite this framework, peripheral nervous system manifestations remain diagnostically challenging because SLE can be associated with diverse neuropathy mechanisms (immune-mediated, inflammatory, ischemic/vasculitic, compressive, metabolic, medication-related), which may overlap clinically and electrophysiologically.

Peripheral nervous system involvement is not rare in SLE cohorts, but immune-mediated demyelinating polyradiculoneuropathies represent a small and clinically high-stakes subset. About 17.7% of patients with SLE have peripheral nervous system (PNS)-SLE syndrome, and amongst these patients, Guillain-Barré syndrome (GBS) is seen in about 1.1% [3]. These data highlight that although demyelinating polyradiculoneuropathies are infrequent in SLE, they may be under-recognized and can carry a disproportionate burden of disability and acute care needs.

Acute inflammatory demyelinating polyneuropathy (AIDP), the classic demyelinating form under the GBS umbrella, is the most severe acute immune-mediated paralytic neuropathy and is associated with a substantial risk of respiratory failure. Standard disease-modifying treatment relies on intravenous immunoglobulin (IVIg) or plasma exchange (PLEX) alongside careful supportive management, reflecting the potentially rapid progression and systemic complications of this disorder [4]. Chronic inflammatory demyelinating polyneuropathy (CIDP), in contrast, follows a chronic or relapsing course and remains a treatable immune-mediated neuropathy; evidence-based guidance recommends IVIg or corticosteroids as initial therapy, with plasma exchange for patients who do not respond adequately to first-line options [5].

When AIDP/GBS or CIDP occurs in the context of SLE, clinicians face additional uncertainties regarding immunopathogenesis, diagnostic interpretation (including the reliability of “classic” cerebrospinal fluid and electrophysiological hallmarks), the relationship to systemic lupus activity, and the extent to which lupus-directed immunosuppression should be added to standard GBS/CIDP treatments. Existing evidence is scattered, with the majority originating from case reports and case series, thereby limiting definitive conclusions. This systematic review aims to synthesize the available clinical literature on SLE-associated AIDP/GBS and CIDP, focusing on clinical presentations, diagnostic modalities, treatment strategies, and outcomes to support earlier recognition and more rational, evidence-informed management of these rare but serious complications.

Due to the infrequency of these manifestations and the lack of established diagnostic or therapeutic protocols, major uncertainties persist concerning the clinical presentation, pathophysiological processes, and appropriate management of SLE-associated inflammatory demyelinating neuropathies. This systematic review seeks to consolidate the existing evidence to enhance the characteristic clinical features, diagnostic methodologies, treatment modalities, and outcomes of AIDP/GBS and CIDP in patients with SLE, thereby addressing deficiencies in the literature.

## Review

Methadology 

Study Design and Reporting Standards

This systematic review was conducted to evaluate the mechanisms, clinical characteristics, and outcomes of immune-mediated demyelinating neuropathies-AIDP and CIDP-associated with SLE. The review was designed and reported in accordance with the Preferred Reporting Items for Systematic Reviews and Meta-Analyses (PRISMA) guidelines.

Literature Search Strategy

A comprehensive and systematic literature search was performed across multiple electronic databases, including PubMed, Embase, Cochrane Library, Scopus, and Google Scholar, to identify relevant studies reporting AIDP or CIDP in the setting of SLE. The search strategy combined controlled vocabulary terms (e.g., MeSH and Emtree terms) and free-text keywords related to systemic lupus erythematosus, lupus, acute inflammatory demyelinating polyneuropathy, Guillain-Barré syndrome(GBS), and chronic inflammatory demyelinating polyneuropathy.

The initial database search yielded 110 records from PubMed, 418 from Embase, 15 from the Cochrane Library, 1,061 from Scopus, and 510 from Google Scholar, resulting in a total of 2,121 records. The complete database-specific search strategies are provided in Appendix 1.

Study Selection

All retrieved records were imported into reference management software, and 368 duplicate records were identified and removed. The remaining 1,753 unique articles underwent primary screening based on titles and abstracts by two independent authors AV and KMA. Studies were screened independently to assess relevance to SLE-associated AIDP or CIDP.

Following primary screening, 154 articles were selected for full-text review. Full texts were assessed for eligibility based on predefined inclusion and exclusion criteria. Eligibility was assessed by three independent authors, AV, KMA, and SB. For discrepancies between the three authors regarding the selection of the articles, authors AT and SK jointly made the final decision of whether to include or exclude the articles. After detailed evaluation, five studies met all eligibility criteria and were included in the final qualitative synthesis.

Eligibility Criteria

Inclusion criteria were: original studies, case series describing AIDP or CIDP in patients with SLE. Studies reporting clinical features, diagnostic findings, proposed immunopathogenic mechanisms, treatment strategies, or outcomes. Articles published in the English language.

Exclusion criteria were: studies involving peripheral neuropathies not consistent with immune-mediated demyelinating pathology. Reviews, case reports, editorials, conference abstracts without sufficient clinical data, or animal studies. Articles lacking clear diagnostic confirmation of SLE or AIDP/CIDP.

Data extraction

**Figure 1:**
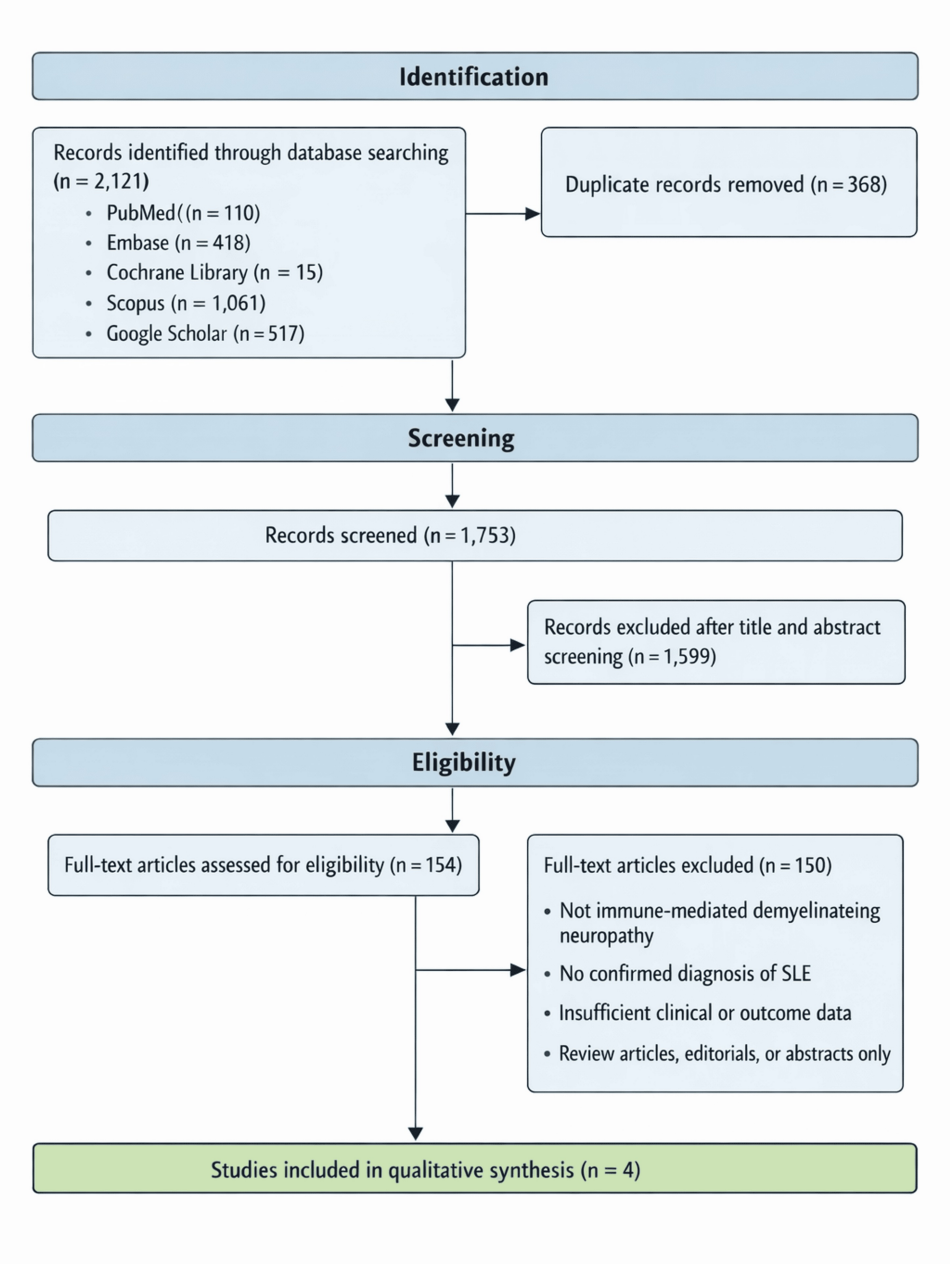
PRISMA Flowchart for selection of the studies

Data were extracted independently from eligible studies using a standardized data collection form. Extracted variables included study design, patient demographics, SLE characteristics, type of demyelinating neuropathy (AIDP or CIDP), diagnostic modalities, therapeutic interventions, and clinical outcomes. Since the sample size was quite small in each study and only four studies were finally included, a meta-analysis or meta-regression cannot be performed. Percentages and ranges were calculated wherever possible to improve the article's readability. The study selection process, as per PRISMA, is reported in Figure 1. 

Results

The extracted data from the four studies are shown below. Table 1 shows studies related to AIDP, and Table 2 shows studies related to CIDP. For AIDP, included studies included work by Xiong et al. [6] and Wang et al. [7]. For CIDP, included studies consisted of work by Wang et al. [7], Vina et al. [8], and Julio et al. [9].

**Table 1:** Studies showing association of AIDP in SLE The most common clinical manifestation was acute limb weakness occurring in approximately 82–83% of patients(all 4 limb weakness: 82.1%, accompanied by sensory disturbances (75%) and hyporeflexia or areflexia (71%), while cranial nerve involvement and respiratory failure were each observed in about 39% of cases. CSF analysis demonstrated albuminocytologic dissociation in 67.9–100% of patients, and electrophysiological studies showed neurogenic or demyelinating abnormalities in the majority of tested individuals; antiganglioside antibodies were largely negative (75% of tested patients). AIDP: acute inflammatory demyelinating polyneuropathy, SLE: systemic lupus erythematosus, GBS: Guillain-Barré syndrome, ANA: antinuclear antibody, LL's: lower limbs, GM: ganglioside monosialic acid, GD: ganglioside disialic acid, DNA: deoxyribonucleic acid, SSA: Sjögren’s syndrome–related antigen A, IVIg: intravenous immunoglobulin, PLEX: plasma exchange, AMAN: acute motor axonal neuropathy, AMSAN: Acute motor and sensory axonal neuropathy, MFS: Miller Fisher syndrome, CSF: cerebrospinal fluid, MPS: methylprednisolone, EMG: electromyography, SLEDAI: systemic lupus erythematosus disease activity index, SEP: somatosensory evoked potentials, SSR: sympathetic skin response, SSB: Sjögren’s Syndrome–related antigen B, dsDNA: double-strand DNA.

Study	Type of study	No of patients	Mean age of patients (years)	Female:Male ratio	Duration of SLE	Clinical features	Diagnostic tests	Treatment modalities	Outcomes
Xiong et al. [6]	Systematic Review	28	31.5	19:09	Not mentioned, 16 (57.1%) had SLE-related GBS before SLE diagnosis.	Four limbs weakness 23 (82.1%), Weakness limited to LLs 5 (17.9%), Sensory disturbances 21 (75.0%), Hyporeflexia or areflexia 20 (71.4%), Cranial nerve involvements 12 (39.3%), Ataxia 2 (7.1%), Dysautonomia 1 (3.6%), Respiratory failure 11 (39.3%), Non-invasive ventilation 4/11 (36.4%), Invasive ventilation 7/11 (63.6%), Malar rash 2 (12.5%), Oral ulcers 1 (6.3%), Arthritis 1 (6.3%), AMAN in 5/27 (18.5%), AMSAN in 5/27 (18.5%), and MFS in 1/27 patients (3.7%).	Albumin-cytological dissociation: Present 19/26 (67.9%), Absent 7/26 (25.0%), Antiganglioside antibodies N/A 20 (71.4%), GM2 and GM3 1/8 (12.5%) GM1 and GD1b 1/8 (12.5%), Negative 6/8 (75.0%), Antinuclear antibody 7 (43.8%), Anti-double-stranded DNA antibody 8 (50.0%), Anti-Smith antibody 0 (0.0%), Anti-SSA antibody 1 (6.3%), Anti-RNP antibody 0 (0.0%), Low complement levels 5 (31.3%).	26 patients received Cyclophosphamide before Cyclophosphamide therapy, 14 patients (50.0%) were treated with IVIg, PLEX, or both (IVIg, 9; PLEX, 1; and both,4).	24 patients (85.7%) reported resolution or improvement after Cyclophosphamide, 1 (3.6%) relapse, 4 (14.3%) showed no improvement after Cyclophosphamide, 1 out of these received Rituximab and improved.
Wang et al. [7]	Retrospective Cohort, Patients treated at Peking Union Medical College Hospital between January 2004 and November 2021	6	32.5 years	2:01	1 week to 36 years, median 5 months. 4/6 (66%) patients had GBS as the primary presentation of SLE. course of GBS, 1 week to 2 months.	5/6 patients had limb weakness, 1 had limb paresthesias and pain, 1 had cardiorespiratory arrest, 1 had generalized tonic clonic seizure, SLEDAI score mean 8, ranged from 2-16. Even mild-moderate SLE activity had GBS development (features of SLE can be added).	All 6 patients had albuminocytological dissociation. A higher CSF protein level was reported as associated with severe disease activity. 1 patient had CSF protein of 7/09gm/L and had no improvement even after all 4 Rx options. CSF Ganglioside was negative in all tested patients. EMG: 4/5 patients had neurogenic lesions, 1/5 had no neurogenic or myogenic lesion but had prolonged F wave latency, 1 had SEP of right lower limb, 1 had SSR in lower limbs on top of a neurogenic lesion. ANA: homogenous pattern in 2patients and speckled pattern in 4 patients. Anti-dsDNA present in 3/6, anti-Smith 1/6, anti-SSA 3/6, anti-SSB 1/6.	6 patients received IVIg. 5 patients received IV MPS, 1 pt received PO Prednisone, 4 patients received Cyclophosphamide. 5 patients received intrathecal Dexamethasone and Methotrexate.	2 pateints had complete resolution.(one with IVIg and Prednionse, 2^nd^ patient after all 4 Rx modalities) 3 patients had marked improvement and 1 had no improvement(all received all 4 Rx modalities).

**Table 2:** : Studies showing association of CIDP in SLE Limb weakness was the predominant clinical manifestation (83–100%), presenting as tetraparesis or proximal limb weakness, with global areflexia (56.3%), sensory disturbances (31–33%), facial paralysis (~33%), and ataxic gait (18.8%). CSF showed albuminocytologic dissociation in 18.8–100% of patients, nerve conduction and electromyography studies commonly showed demyelinating abnormalities such as slowed conduction velocities (68.8–100%), absent or prolonged F-waves (37.5–66.7%), conduction blocks (~12–33%), and reduced sensory responses (up to 100%). Antiganglioside antibodies were generally negative in the tested patients. CIDP: chronic inflammatory demyelinating polyneuropathy, SLEDAI: systemic lupus erythematosus disease activity index, SLE: systemic lupus erythematosus, EMG: electromyography, ANA: antinuclear antibody, RNP: ribonucleoprotein, dsDNA: double-strand deoxyribonucleic acid,  U1RNP: U1 small nuclear ribonucleoprotein, AMA-M2: anti-mitochondrial M2 antibody, NCS: nerve conduction study, ESR: erythrocyte sedimentation rate, WBC: white blood cell, CRP: C-reactive protein.

Study	Type of study	No of patients	Mean age of patients (years)	Female: Male ratio	Duration of SLE	Clinical features	Diagnostic tests
Wang et al. [7]	Retrospective Cohort, Patients treated at Peking Union Medical College Hospital between January 2004 and November 2021	3	50.66 years	All 3 females	1 week to 36 years, median 5 months, course of CIDP 2 months to 15 months.	2 had weakness in proximal limbs, 1 had limb weakness (non-specified), 1 had facial paralysis, and 1 had stocking glove paresthesia. Even mild-moderate SLEDAI score pts had CIDP development. (Features of SLE can be added).	All 3 patients had albuminocytological dissociation. CSF Ganglioside was negative in all tested patients. EMG: Neurogenic changes in all 3, Myogenic changes in 1, Motor conduction block in 1 patient. ANA: Speckled pattern in 2 patients and homogenous pattern in 1 patient. Anti-Sm, anti-RNP in 1 patient, Anti-dsDNA, in 1 patient, 1 unavailable anti-SSA, anti-U1RNP, AMA-M2, antinucleosome, antihistone.
Vina et al. [8]	Case Series and Systematic Review	6	45.66 years	100% patients were females	2/6 had CIDP as the initial presentation. The onset of CIDP from SLE diagnosis in 4/6 patients was 1 year to 9 years.	1/6 patients had predominantly bilateral lower limb weakness, 5/6 had all 4 limb weakness, and 2/5 patients had no discrepancy in weakness of arms and legs. (Reflexes to be added).	ANA titer ranged from 1:640 to 1:1280. Motor Nerve Involvement: Reduced amplitudes in motor nerves: 100% (6/6) Slowed conduction velocities: 100% (6/6) Prolonged distal latencies: 100% (6/6) Conduction blocks: 33% (2/6; peroneal and median nerves) Absent or borderline F-waves: 66.7% (4/6) Sensory Nerve Involvement: Absent or reduced sural, median, or ulnar sensory responses: 100% (6/6) EMG Findings: Neurogenic changes: 100% (6/6) Myopathic features: 16.7% (1/6)
Julio et al. [9]	Systematic Review	19 patient (16 from the literature review, 3 from their own)	34.6 years	13:3	Previous diagnosis of SLE in 5 pts(31.25%), concomittant in 7 pts(43.7%), and initial CIDP in 4 pts(25%).	Common clinicl features were tetraparesis in 8(50%), global areflexia 9(56.3%), hypoaesthesia in 5(31.25%), parapareis 6(37.5%), ataxia gait 3(18.75%), hyporeflexia 3(18.8%). At diagnosis of CIDP, 10(62.5%) had concomittant SLE activity, 6(37.5%) were in remission.	Albuminocytological dissociation seen in 3(18.8%) pts. NCS+EMG showed slowing of conduction velocities 11(68.75%), absence of F waves 6(37.5%), demyelinating polyradiculopathy in 4(25%), absent sensory potential in 4(25%), motor nerve conduction block 3(12%), reduced amplitude of compound muscle action potential 2(12.5%), absence of motor nerve responses 2(12.5%) . ANA positive in 100%, dsDNA 10(62.5%), anti Sm 6(37.5%), anti Ro 5(31.25%), anti RNP 4(25%), anticardiolipin 5(31.25%). Most common features of active SLE were complement consumption in 3(18.75%),proteinuria 3(18.8%), leukopenia 2(12.5%), hematuria, lymphopenia 2(12.5%). Increased ESR 4(25%), increased WBC count, increased CRP, and hypergammaglobulinemia.

Epidemiology

**Figure 2:**
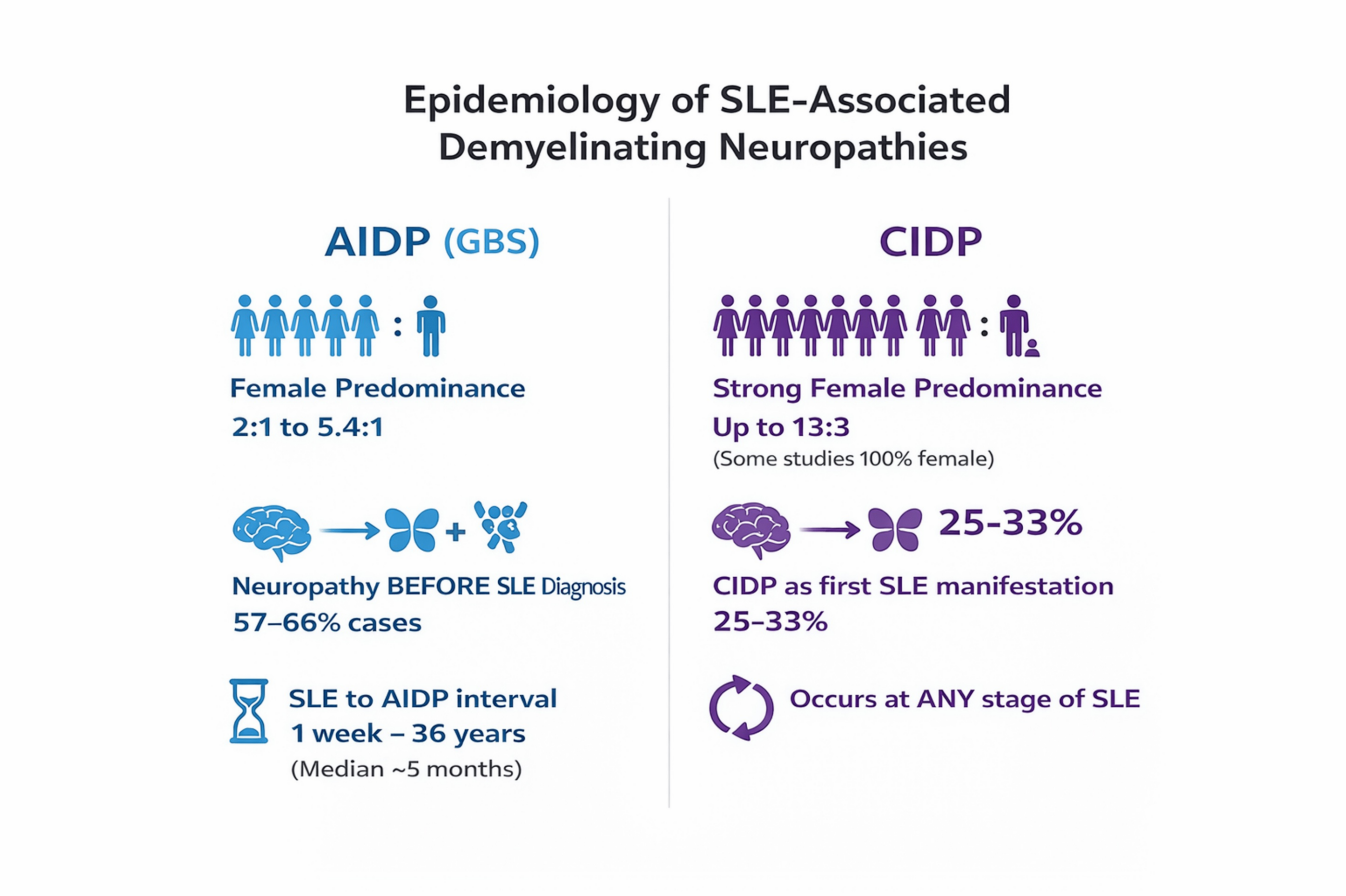
Female:Male ratio and temporal pattern of SLE Figure created by the authors based on data extracted from references [6,7,8,9]. SLE: systemic lupus erythematosus, AIDP: acute inflammatory demyelinating polyneuropathy, GBS: Guillain-Barré syndrome, CIDP: chronic inflammatory demyelinating polyneuropathy.

**Figure 3:**
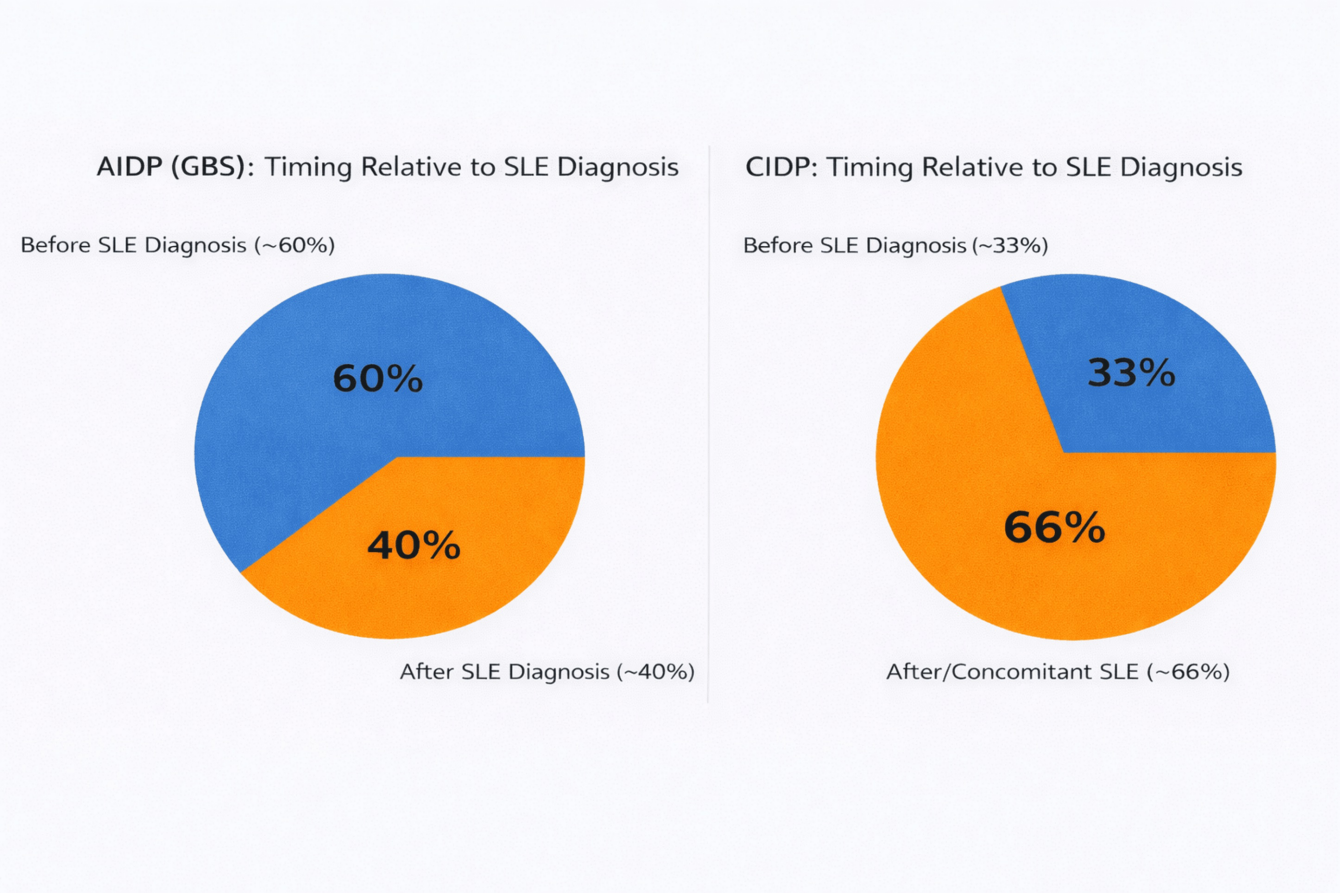
AIDP vs CIDP timing relative to SLE diagnosis Figure created by the authors based on data extracted from references [6,7,8,9]. AIDP: acute inflammatory demyelinating polyneuropathy, GBS: Guillain-Barré syndrome, CIDP: chronic inflammatory demyelinating polyneuropathy, SLE: systemic lupus erythematosus.

Out of the four studies included for AIDP, two studies were cross-sectional studies, one study was a retrospective cohort and one study was a systematic review. For AIDP, the female-to-male ratio ranged from approximately 2:1 to as high as 5.4:1. For CIDP, two studies had exclusively female patients (100% female), while one study showed a strong female-to-male ratio of 13:3. SLE-related AIDP occurrence before SLE was formally diagnosed from 57.1-66%. This shows that in more than half of reported cases, SLE-related AIDP occurred before SLE was formally diagnosed. In patients with SLE as the primary event, the interval between SLE onset and GBS ranged widely, from as early as one week to as long as 36 years, with a median duration of approximately five months. Regarding the temporal association of SLE and CIDP, 25%-33% had CIDP as the initial manifestation of SLE, 43.7% presented with concomitant SLE and CIDP, and 31.25% of patients had a prior diagnosis of SLE before developing CIDP. Overall, these data indicate that CIDP may occur at any stage of SLE, with a substantial proportion presenting either concomitantly or as the first clinical manifestation, emphasizing the importance of considering underlying SLE in patients presenting with CIDP. The duration of systemic lupus erythematosus (SLE) in patients with CIDP was highly variable, ranging from one week to 36 years, with a median duration of five months. Figure 2 summarizes the epidemiology of SLE-associated demyelinating neuropathies. Figure 3 summarizes the temporal relation of SLE with AIDP and CIDP.

Clinical Features

For AIDP, the study by Xiong et al., consisting of 28 patients, showed that the majority of patients presented with four-limb weakness (82.1%), while 17.9% had weakness limited to the lower limbs. Sensory disturbances were common (75.0%), along with hyporeflexia or areflexia (71.4%). Cranial nerve involvement and respiratory failure were each observed in 39.3% of patients. Among those with respiratory failure, 36.4% required non-invasive ventilation, and 63.6% required invasive mechanical ventilation. Less frequent neurological features included ataxia (7.1%) and dysautonomia (3.6%). Manifestations of SLE were malar rash (12.5%), oral ulcers (6.3%), and arthritis (6.3%). Regarding electrophysiological subtypes, Acute motor axonal neuropathy (AMAN) and Acute motor and sensory axonal neuropathy (AMSAN) each accounted for 18.5%, while Miller Fisher syndrome (MFS) was identified in 3.7% of cases. The study by Wang et al. showed that 83.3% (5/6) of patients presented with limb weakness, while 16.7% presented with limb paresthesias and pain. Cardiorespiratory arrest (16.7%) and generalized tonic-clonic seizures (16.7%) were seen as well. The mean SLEDAI score was 8 (range 2-16), indicating that Guillain-Barré syndrome can occur even in patients with mild to moderate SLE activity. Overall, these findings highlight predominant motor involvement with frequent sensory deficits and a substantial burden of respiratory complications, despite often modest systemic lupus activity.

**Figure 4:**
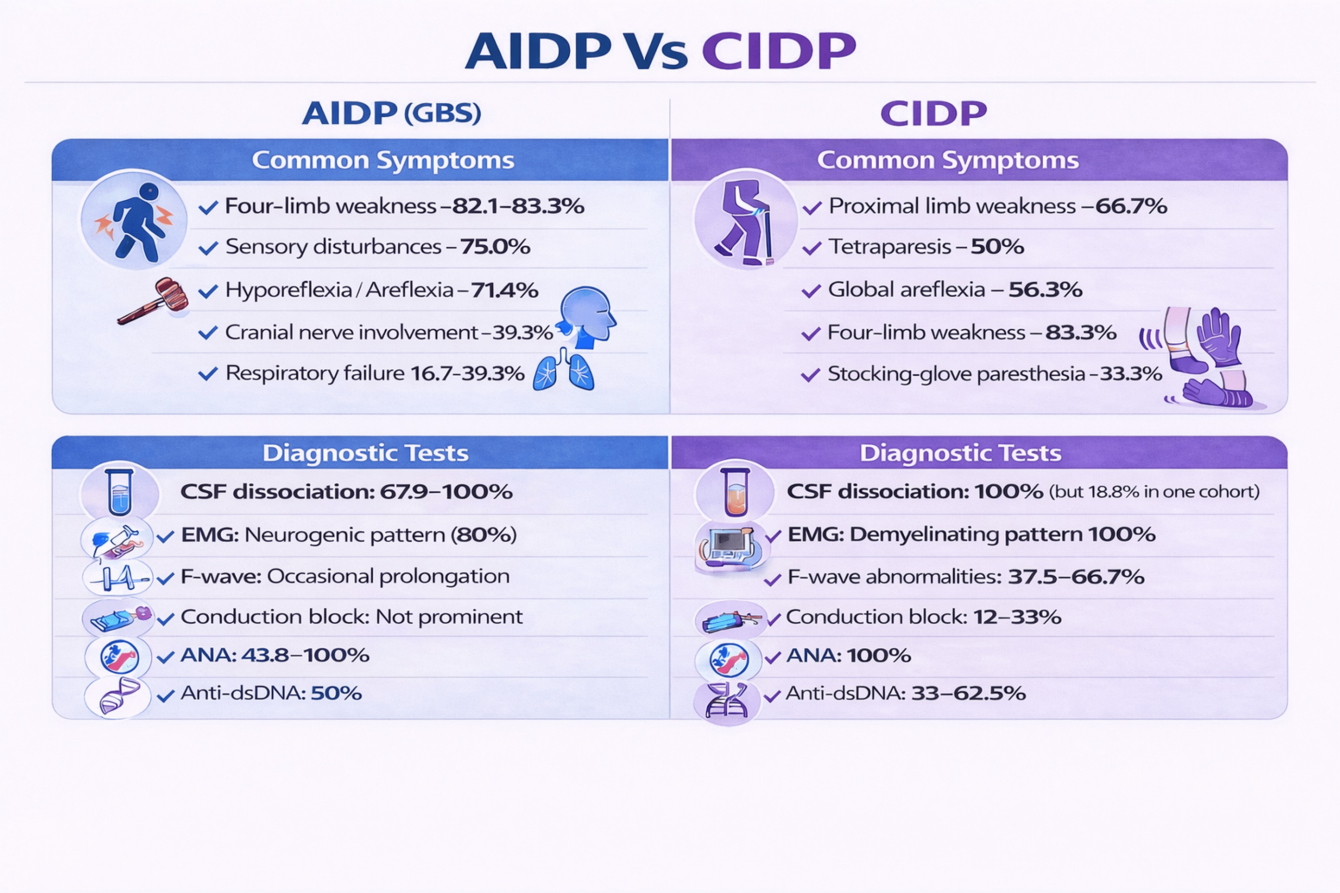
Clinical and diagnostic features Figure created by the authors based on data extracted from references [6,7,8,9]. AIDP: acute inflammatory demyelinating polyneuropathy, CIDP: chronic inflammatory demyelinating polyneuropathy, GBS: Guillain-Barré syndrome, CSF: cerebrospinal fluid, EMG: electromyography, ANA: antinuclear antibody, Anri-dsDNA: .

Regarding CIDP, the study by Wang et al. showed that proximal limb weakness was observed in 66.7%, while non-specified limb weakness occurred in 33.3%. Facial paralysis was present in 33.3%, and stocking-glove paresthesia was reported in 33.3%. The study by Vina et al. showed that, among six patients, 83.3% had weakness affecting all four limbs, while 16.7% had predominantly bilateral lower limb weakness. Of the five patients with all four-limb involvement, 40% showed no discrepancy in the severity of weakness between arms and legs. The study by Julio et al. showed that the most common neurological manifestations were tetraparesis in 50% and global areflexia in 56.3% of patients. Other features included paraparesis in 37.5%, hypoaesthesia in 31.25%, ataxic gait in 18.75%, and hyporeflexia in 18.8%. At the time of CIDP diagnosis, 62.5% of patients had active SLE, while 37.5% were in remission. Figure 4 summarizes common symptoms and diagnostic tests for AIDP and CIDP related to SLE.

Lab Findings

In AIDP pts, the study by Xiong showed that cerebrospinal fluid (CSF) (n=26) was tested: Albumin-cytological dissociation was present in 67.9% of patients and absent in 25.0%. Antiganglioside antibodies (n=8 tested): GM2/GM3 positivity in 12.5%, GM1/GD1b positivity in 12.5%, and negative in 75.0%. The remaining 20 pts were not tested. Antinuclear antibody (ANA) was positive in 43.8%, anti-double-stranded DNA in 50.0%, anti-Sjögren’s syndrome-related antigen A (SSA) in 6.3%, and anti-Smith and anti- ribonucleoprotein (RNP) were negative in all tested patients. Complement levels: Low complement was observed in 31.3% of patients. According to the study by Wang et al., CSF findings showed that all patients (100%) had albuminocytological dissociation. Higher CSF protein levels were associated with more severe disease; one patient with a CSF protein of 7.09 g/L showed no improvement despite receiving all four treatment options. CSF ganglioside antibodies were negative in all tested patients. Electrophysiology (EMG/other studies): 80% had neurogenic lesions. One patient had neither neurogenic nor myogenic lesions but demonstrated prolonged F-wave latency. Additional studies showed somatosensory evoked potential (SEP) abnormality in the right lower limb in one patient and sympathetic skin response (SSR) abnormalities in the lower limbs in another, alongside neurogenic lesions. ANA was positive in all six patients, with a homogeneous pattern in 33.3% and a speckled pattern in 66.7%. Anti-double-strand DNA (dsDNA) was present in 50%, anti-Smith in 16.7%, anti-SSA in 50%, and anti- Sjögren’s syndrome-related antigen B (SSB) in 16.7%. 

Regarding CIDP, the study by Wang et al. showed CSF findings where all patients (100%) had albumin-cytological dissociation. CSF ganglioside antibodies were negative in all tested patients. In electrophysiology (EMG), all three patients showed neurogenic changes. Myogenic changes and motor conduction block were each observed in one patient (33.3%). ANA was positive in all patients in autoantibodies, with a speckled pattern in 66.7% and a homogeneous pattern in 33.3%. Anti-Smith and anti-RNP were present in one patient (33.3%), anti-dsDNA in one patient (33.3%), and data were unavailable for one patient. Other antibodies tested included anti-SSA, anti-U1 small nuclear ribonucleoprotein (U1RNP), anti-mitochondrial M2 antibody (AMA-M2), antinucleosome, and antihistone, though individual positivity was not specified. The study by Vina et al. showed that ANA titer ranged from 1:640 to 1:1280. Motor nerve involvement showed reduced amplitudes in motor nerves: 100% (6/6), slowed conduction velocities: 100%, prolonged distal latencies: 100%, conduction blocks: 33%, absent or borderline F-waves: 66.7%, sensory Nerve Involvement, absent or reduced sural, median, or ulnar sensory responses: 100%, EMG findings: neurogenic changes: 100%, myopathic features: 16.7%. The study by Julio et al. showed that CSF Findings: Albumin-cytological dissociation: 18.8%. Electrophysiology [Nerve conduction study (NCS) + EMG]: Slowing of conduction velocities: 68.75%, Absent F-waves: 37.5%, Demyelinating polyradiculopathy: 25%, Absent sensory potentials: 25%, Motor nerve conduction block: 12%, Reduced compound muscle action potential (CMAP) amplitude: 12.5%, Absent motor nerve responses: 12.5%, Autoantibodies: ANA positive: 100%, Anti-dsDNA: 62.5%, Anti-Smith: 37.5%, Anti-Ro/SSA: 31.25%, Anti-RNP: 25% (4/16), Anticardiolipin: 31.25%, Active SLE Features at CIDP Presentation: Complement consumption: 18.75%, Proteinuria: 18.8%, Leukopenia: 12.5%, Hematuria / Lymphopenia: 12.5%, Increased ESR: 25%, Increased WBC, CRP, hypergammaglobulinemia: observed (exact numbers not specified).

Treatment and outcomes

For management of AIDP in SLE, as reported by Xiong et al., cyclophosphamide was the predominant immunosuppressive therapy, administered to 92.9% (26/28) of patients. Before cyclophosphamide initiation, 50.0% (14/28) received first-line immunomodulatory treatments, including IVIg alone in 32.1% (9/28), plasmapheresis alone in 3.6% (1/28), and combined IVIg plus plasmapheresis in 14.3% (4/28). Following cyclophosphamide therapy, 85.7% (24/26) experienced clinical resolution or improvement, while 3.6% (1/28) had disease relapse. 14.3% (4/28) showed no improvement after cyclophosphamide; notably, one non-responder subsequently received rituximab and demonstrated clinical improvement. In the study by Wang et al., all patients (100%, 6/6) received IVIg. Adjunctive immunosuppressive therapy was frequently used, including intravenous methylprednisolone in 83.3% (5/6) and oral prednisone in 16.7% (1/6). Additional treatments comprised cyclophosphamide in 66.7% (4/6) and intrathecal dexamethasone with methotrexate in 83.3% (5/6). Regarding outcomes, 33.3% (2/6) achieved complete resolution, one with IVIg and prednisone alone, and one after receiving all four treatment modalities. 50% (3/6) demonstrated marked clinical improvement. These patients received MPS and Cyclophosphamide.16.7% (1/6) showed no improvement even after receiving all four treatment modalities. 

**Figure 5:**
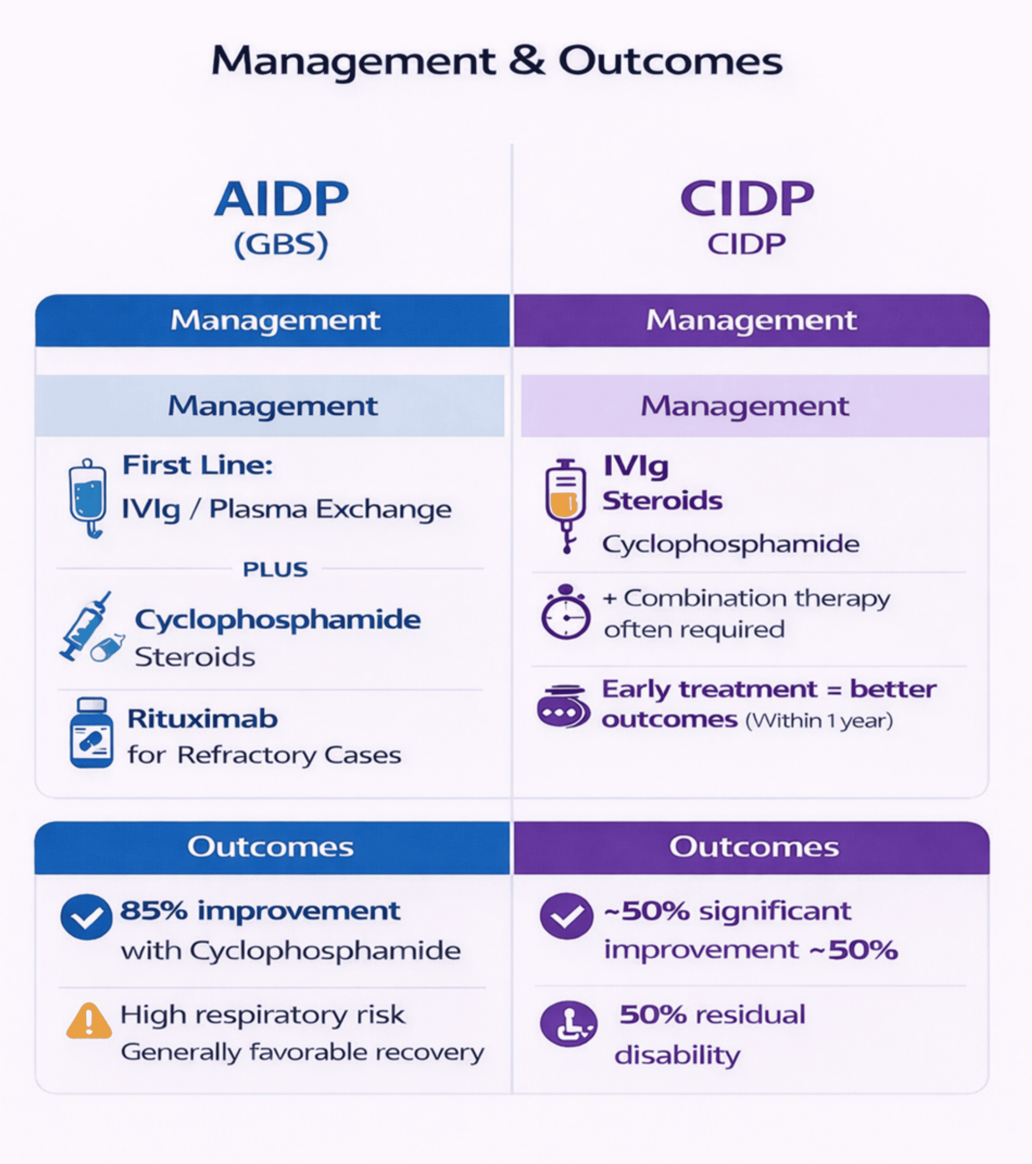
Management and outcomes of AIDP and CIDP in SLE Figure created by the authors based on data extracted from references [6,7,8,9]. AIDP: acute inflammatory demyelinating polyneuropathy, CIDP: chronic inflammatory demyelinating polyneuropathy, GBS: Guillain-Barré syndrome, IVIg: intravenous immunoglobulin.

For management of CIDP in SLE, in the study by Wang et al., all patients 100% (3/3) received IVIg, oral prednisone, and cyclophosphamide, with 66.7% (2/3) additionally treated with intrathecal dexamethasone and methotrexate. Clinical outcomes consisted of 66.7% (2/3) demonstrating marked improvement, while 33.3% (1/3) showed no improvement despite intrathecal treatment. The two patients who showed marked improvement received Methylprednisolone, Cyclophosphamide, and one of them received intrathecal Dexamethasone and Methotrexate as well. In the cohort reported by Vina et al., all patients (6/6) received corticosteroids via oral and/or intravenous routes. IVIg was administered to 66.7% (4/6) at 0.4 g/kg/day for three to five days, with 50% (3/6) subsequently receiving monthly IVIg for two to seven months; 16.7% (1/6) of these received weekly IVIg for eight weeks. PLEX was used in 50% (3/6), primarily in patients with incomplete or refractory responses to IVIg. Clinically, 50% (3/6) achieved >50% symptomatic improvement, while the remaining 50% (3/6) showed minimal improvement. Favorable response was associated with early IVIg initiation (within one year of CIDP diagnosis; mean seven months) and pre-existing SLE before CIDP onset. In contrast, poor response correlated with delayed IVIg treatment (mean 60 months after symptom onset) and CIDP preceding SLE diagnosis in 66.7% (2/3) of poor responders. In the study by Julio et al., the majority of patients, 81.25% (13/16), required combination immunomodulatory therapy with more than two agents. Corticosteroids were the most frequently used treatment, 75% (12/16), followed by IVIg (68.75%, 11/16) and PLEX (37.5%, 6/16). Additional immunosuppressive therapies included cyclophosphamide (31.25%, 5/16), azathioprine (12.5%, 2/16), cyclosporine (12.5%, 2/16), mycophenolate mofetil (6.25%, 1/16), and rituximab (6.25%, 1/16). Despite intensive treatment, outcomes were heterogeneous: 50% (8/16) achieved significant clinical improvement, whereas 50% (8/16) continued to have major residual disabilities. Figure 5 summarizes the management and outcomes of AIDP and CIDP associated with SLE.

Discussion

This systematic review collects data from two studies on Acute Inflammatory Demyelinating Polyradiculoneuropathy (AIDP) and three studies on CIDP with the context of SLE. The findings highlight that while these neuropathies are rare manifestations of SLE, they possess distinct demographic, clinical, and prognostic features that differentiate them from their idiopathic counterparts.

Distinct Demographic and Temporal Patterns

A defining feature of SLE-associated polyneuropathies is the profound female predominance. While idiopathic GBS typically shows a slight male preponderance, the included studies reveal female-to-male ratios as high as 5.4:1 for AIDP and 13:3 for CIDP, with some studies consisting exclusively of females. This reflects the underlying epidemiology of lupus and serves as an important diagnostic clue.

The temporal data suggest that inflammatory neuropathy frequently acts as the "herald" manifestation of lupus. In AIDP, about 57.1% to 66% of cases, neuropathy occurred before the formal diagnosis of SLE. In CIDP, approximately one-third of patients presented with neuropathy as their initial SLE manifestation.

Implication: This challenges the traditional view of neurological involvement occurring only in established disease. Clinicians should maintain a high index of suspicion for underlying autoimmunity in all patients, especially young female patients presenting with "idiopathic" demyelinating neuropathy, even in the absence of classic malar rash or arthritis and other SLE features.

Clinical Severity and Systemic Disease Activity

The review identifies a notable dissociation between systemic lupus activity and neurological severity. In the AIDP cohorts, the mean SLEDAI score was 8 (indicating mild-to-moderate activity), yet the neurological burden was severe, with nearly 40% of patients experiencing respiratory failure, a rate significantly higher than the 20-30% typically cited in idiopathic GBS [10,11].

Similarly, in CIDP, 37.5% of patients were in systemic remission at the time of neuropathy diagnosis. This indicates that neuro-inflammation may proceed independently of other organ damage (e.g., nephritis or dermatitis), suggesting that low systemic disease activity should not preclude aggressive neurological workup or treatment.

Pathophysiological Insights: The Autoantibody Divergence

The diagnostic profile of SLE-associated AIDP differs from classic post-infectious GBS.

Anti-ganglioside Antibodies: These antibodies (e.g., GM1, GD1a), which drive the mechanism of molecular mimicry in idiopathic GBS, were positive in about 12.5% of the patients in studies. This is in conjunction with idiopathic AMAN and AIDP variants of GBS, where 60% of AMAN patients had anti-GD1a antibodies positive, as compared to only 4% of AIDP patients having anti-GD1a antibodies positive [12]. For ANA & Anti-dsDNA, high positivity rates (up to 100% for ANA) were observed.

For SLE-associated CIDP, the etiopathology consists of immune complex deposition in the endoneurial and epineurial blood vessel wall supplying the nerves and Schwann cell basement membrane [13]. In contrast, the mechanism of idiopathic CIDP is different, where there is coordinated humoral and cellular immune responses directly targeting the myelin of peripheral nerves [14]. Albuminocytological dissociation reflects the breakdown of blood nerve barriers at nerve roots and distal terminals, where demyelination is most prominent, whereas in SLE-associated CIDP, there is immune complex deposition in vascular walls and vasculopathy-mediated ischemic nerve damage, and hence, albuminocytological dissociation is not prominent [13,15]. The serological profile findings in the included studies agree with this proposed mechanism. Consequently, the CSF findings were variable; while albumin-cytological dissociation is the hallmark of idiopathic GBS and CIDP, it was absent in a significant proportion of SLE-CIDP patients (only 18.8% positive in the Julio et al. cohort), warning clinicians that normal CSF protein does not rule out lupus-related CIDP, and normal CSF protein in CIDP should prompt physicians to think of SLE as the probable underlying cause of CIDP.

Therapeutic Strategies and the "Window of Opportunity"

The management data underscores the necessity of immunosuppression over simple immunomodulation. IVIg and plasma exchange remain the cornerstone therapies for Guillain-Barré syndrome, with studies describing variable clinical course and recovery patterns [16,17].

AIDP Management: While IVIg and plasmapheresis remain first-line, the study by Anji Xiong et al. highlights the pivotal role of Cyclophosphamide, showing an 85.7% response rate. The study by Wang et al. also highlights 83.33% response rate with combined MethylPrednisolone and Cyclophosphamide treatment. This suggests that, unlike idiopathic GBS, which is self-limiting with immunomodulation, SLE-AIDP requires immunosuppressive therapy on top of immunomodulatory therapy to halt the underlying autoimmune drive and reduce the ongoing production of the antibodies causing the disease.

CIDP Management: Standard treatment of idiopathic CIDP consists of IVIg or corticosteroids, with PLEX reserved for refractory cases. [5] CIDP associated with SLE requires far more aggressive immunosuppression compared to standard CIDP therapies, with the addition of immunosuppressive drugs like cyclophosphamide, azathioprine, mycophenolate, and Rituximab. [18,19] Most patients in the studies included in our review received one of the above medications during the management course. Outcomes for CIDP were more heterogeneous, with about 50% residual disability rate even after receiving multiple combination treatment therapies as per Julio et al. The data from Vina et al. provides a crucial prognostic factor - time to treatment. A favorable outcome was associated with IVIg initiation within 1 year. A poor outcome was associated with delayed treatment.

This defines a critical "window of opportunity" where early aggressive therapy-often combining IVIg/corticosteroids with maintenance immunosuppression (cyclophosphamide, mycophenolate), can prevent irreversible axonal loss.

Limitations

These findings are limited by the retrospective nature of the included studies and small sample sizes because of rare complications. The variability in treatment protocols (e.g., the use of intrathecal chemotherapy in one cohort) also complicates direct comparisons.

## Conclusions

AIDP and CIDP in SLE represent a distinct clinical entity characterized by a herald presentation, severe respiratory depression risk, and a disconnect from systemic disease activity. The absence of typical anti-ganglioside antibodies and the absence of typical albuminocytological dissociation support a unique autoimmune pathogenesis for demyelination different from the idiopathic AIDP and CIDP mechanisms. Use of Cyclophosphamide and steroids as immunosuppression on top of PLEX and IVIg as immunomodulators for SLE-related AIDP and CIDP should be strongly considered because of the ongoing process of antibody production. Early recognition and the prompt initiation of combination immunotherapy are essential, particularly in CIDP, to avert permanent disability. We strongly suggest future research endeavors on multicenter, potentially multinational registries or retrospective/prospective cohort studies for further research data. Nested case control studies within large SLE cohorts can also help identify risk factors for developing demyelinating neuropathy and an autoantibody profile.

## Appendices

Appendix 1

Detailed Database Search Strategies

1.      PubMed Search

( ( "Systemic Lupus Erythematosus"[Mesh] OR systemic lupus erythematosus[tiab] OR SLE[tiab] OR lupus[tiab] )

 AND

 ( "Guillain-Barre Syndrome"[Mesh] OR guillain-barre[tiab] OR guillain barre[tiab] OR AIDP[tiab] OR acute inflammatory demyelinating polyneuropathy[tiab] OR CIDP[tiab] OR chronic inflammatory demyelinating polyneuropathy[tiab] OR chronic inflammatory demyelinating neuropathy[tiab] ))

AND

( adult[Mesh] OR adult[tiab] OR "18 years"[tiab] )

2.      Embase Search

('systemic lupus erythematosus'/exp OR 'systemic lupus erythematosus':ti,ab OR SLE:ti,ab OR lupus:ti,ab)

AND

('guillain barre syndrome'/exp OR 'acute inflammatory demyelinating polyneuropathy'/exp

OR AIDP:ti,ab

OR 'chronic inflammatory demyelinating polyneuropathy'/exp

OR CIDP:ti,ab)

AND

('adult'/exp OR adult*:ti,ab)

3.      Cochrane Library

("systemic lupus erythematosus" OR SLE OR lupus)

AND

("guillain barre syndrome" OR AIDP OR "acute inflammatory demyelinating polyneuropathy"

OR "chronic inflammatory demyelinating polyneuropathy" OR CIDP)

4.      Scopus

(TITLE-ABS-KEY("systemic lupus erythematosus" OR SLE OR lupus))

AND

(TITLE-ABS-KEY("guillain-barre syndrome" OR AIDP

OR "acute inflammatory demyelinating polyneuropathy"

OR CIDP OR "chronic inflammatory demyelinating polyneuropathy"))

5.      Google Scholar

"systemic lupus erythematosus" AND (AIDP OR CIDP OR "demyelinating polyneuropathy" OR "Guillain Barre")
